# Summary of the National Advisory Committee on Immunization (NACI) Supplemental Guidance on Influenza Vaccination in Adults 65 Years of Age and Older

**DOI:** 10.14745/ccdr.v50i11a02

**Published:** 2024-11-07

**Authors:** Pamela Doyon-Plourde, Angela Sinilaite, Jesse Papenburg

**Affiliations:** 1 Centre for Immunization Programs, Public Health Agency of Canada, Ottawa, ON; 2 NACI Influenza Working Group Chair; 3 Division of Pediatric Infectious Diseases, Department of Pediatrics, Montréal Children’s Hospital of the McGill University Health Centre, Montréal, QC; 4 Division of Microbiology, Department of Clinical Laboratory Medicine, OPTILAB Montréal - McGill University Health Centre, Montréal, QC; 5 Department of Epidemiology, Biostatistics, and Occupational Health, School of Population and Global Health, McGill University, Montréal, QC

**Keywords:** NACI, National Advisory Committee on Immunization, guidance, influenza, influenza vaccine, older adults

## Abstract

**Background:**

Adults 65 years of age and older are at higher risk of influenza complications, such as hospitalization and death. As a result, seasonal influenza immunization is particularly important for this group.

**Objective:**

This supplemental statement provides an evidence summary on the preferential use of one or more of the age-appropriate influenza vaccines for adults 65 years of age and older, over other age-appropriate influenza vaccines.

**Methods:**

The National Advisory Committee on Immunization (NACI)’s Influenza Working Group undertook an overview of existing systematic reviews on the efficacy, effectiveness, safety and cost effectiveness of influenza vaccination in adults 65 years of age and older. Additionally, NACI’s evidence-based process was used to assess the quality of eligible studies, summarize and analyze the findings and apply an ethics, feasibility and acceptability lens to develop recommendations.

**Results:**

The evidence suggests that high-dose inactivated influenza vaccine (IIV-HD), adjuvanted inactivated influenza vaccine (IIV-Adj) and recombinant influenza vaccine (RIV) offer increased benefits for adults 65 years of age and older when compared to standard dose influenza vaccines. The IIV-HD had the most supporting evidence, followed by IIV-Adj and then RIV. Evidence comparing these enhanced vaccines was limited.

**Conclusion:**

Following a thorough review of the complete body of evidence, NACI recommends that IIV-HD, IIV-Adj or RIV should be offered over other influenza vaccines for adults 65 years of age and older. NACI also continues to strongly recommend the inclusion of adults 65 years of age and older among those for whom it is particularly important to receive influenza vaccination.

## Introduction

Adults 65 years of age and older are at higher risk of severe influenza infection and related complications such as pneumonia, hospitalization and death. This risk is significantly higher with increasing age, presence and severity of chronic medical conditions and higher levels of frailty ( ((1–4))). Considering the burden of disease in this population, the National Advisory Committee on Immunization (NACI) has identified adults 65 years of age and older as one of the groups at higher risk of influenza complications for whom influenza immunization is particularly important **(*Strong NACI Recommendation*)** ( ((5))).

NACI has conducted several reviews over the years to evaluate the best available scientific and clinical evidence to develop recommendations for the use of influenza vaccines, with a focus on optimizing influenza protection among older adults in Canada ( ((6,7))).

Other than a recommendation for using high-dose inactivated influenza vaccines (IIV-HD) over standard-dose inactivated influenza vaccine (IIV-SD) formulations, NACI has not previously made comparative individual-level recommendations on the use of other available vaccines in this age group. On a public health program level, NACI has recommended that any of the available influenza vaccines authorized in this age group should be used, as there was insufficient evidence on the incremental value of different influenza vaccines to make comparative public health program-level recommendations on the use of available vaccines.

Given the expressed desire by provincial and territorial programs for guidance on optimal product choice(s) for older adults, NACI has undertaken a review of evidence to determine whether any one or more of the age-appropriate influenza vaccines for adults 65 years of age and older should be preferentially used over other age-appropriate influenza vaccines. A systematic review of economic literature was also undertaken to inform public health program decision-making.

## Methods

The NACI Influenza Working Group undertook an overview of existing systematic reviews to answer the following research question: Should any age-appropriate influenza vaccine(s) be preferentially used in adults 65 years of age and older? The literature search and data extraction were conducted according to the following population, intervention, comparator and outcomes (PICO) framework (**Table 1**).

**Table 1:** Population, intervention, comparator(s), outcome(s) criteria guiding National Advisory Committee on Immunization’s evidence review^a^

PICO	Criteria
Population	Adults 65 years of age and older
Intervention	Inactivated influenza vaccine (IIV)-not standard (not-SD) and recombinant influenza vaccines: · High-dose inactivated influenza vaccine (IIV-HD) · MF-59 adjuvanted inactivated influenza vaccine (IIV-Adj) · Recombinant influenza vaccine (RIV) · Mammalian cell culture-based vaccine (IIV-cc)
Comparator	Inactivated standard-dose influenza vaccines (IIV-SD), inactivated influenza vaccine (IIV)-not SD and RIVs
Outcomes^b^	Vaccine efficacy/effectiveness: · Lab-confirmed influenza (LCI) · Influenza-associated outpatient/emergency department (ED) visits (LCI, influenza-like illness [ILI]) · Influenza-associated hospitalization (LCI, ILI) · Influenza-associated vascular events Vaccine safety: · Any solicited systemic adverse reaction grade ≥3 · Guillain-Barré Syndrome (GBS) · Any serious adverse event (SAE) · Any solicited injection site adverse reaction grade ≥3 Economics: · Vaccine cost effectiveness (cost per life year saved, cost per influenza case averted) · Cost-utility (cost per quality-adjusted life year [QALY])

Abbreviations: ED, emergency department; GBS, Guillain-Barré Syndrome; IIV, inactivated influenza vaccine; IIV-Adj, adjuvanted inactivated influenza vaccine; IIV-cc, mammalian cell culture-based inactivated influenza vaccine; IIV-HD, high-dose inactivated influenza vaccine; IIV-SD, standard-dose inactivated influenza vaccine; ILI, influenza-like illness; LCI, laboratory-confirmed influenza; PICO, population, intervention, comparator and outcomes framework; QALY, quality-adjusted life year; RIV, recombinant influenza vaccine; SAE, serious adverse event

^a^ Table adapted from *NACI Supplemental guidance on influenza vaccination in adults 65 years of age and older* ( ((8)))

^b^ Critical/important outcomes for decision-making

The GRADE-ADOLOPMENT process was employed to adapt recommendations from the United States Advisory Committee on Immunization Practices guideline panel where they assessed the relative benefits and harms of IIV-HD, adjuvanted inactivated influenza vaccines (IIV-Adj) and recombinant influenza vaccine (RIV) compared to one another and with IIV-SD in adults 65 years of age and older ( ((9,10))). Evidence synthesis on the efficacy and cost effectiveness of influenza vaccines in adults 65 years of age and older was further expanded with two additional systematic reviews, both developed in collaboration with the Methods and Applications Groups for Indirect Comparisons through the Drug Safety and Effectiveness Network and supervised by the NACI Influenza Working Group. One review examined the efficacy of influenza vaccines in older adults, while the second review delved into the cost effectiveness of seasonal influenza vaccines in older adults. Further details regarding the methodologies employed in both Drug Safety and Effectiveness Network reviews were published in pre-specified protocols ( ((11,12))).

To support this work, a systematic assessment of ethics, equity, feasibility and acceptability of influenza vaccine guidance was conducted according to established NACI methods ( ((13))). The NACI evidence-based process was used to assess the available evidence and develop updated recommendations ( ((14))). Details and results can be found in the *NACI supplemental guidance on influenza vaccination in adults 65 years of age and older* ( ((8))).

## Results

NACI’s evidence base encompassed an overview of three systematic reviews and meta-analyses to determine if certain authorized age-appropriate influenza vaccines are better suited for adults 65 years of age and older compared to others, analyzing findings from a total of 57 unique primary studies ( ((10,15,16))). Based on the available evidence, NACI concluded that IIV-HD, IIV-Adj and RIV offer greater benefits when compared to IIV-SD, while maintaining the same level of safety (**Table 2**). Additionally, IIV-HD and IIV-Adj also appear to be cost-effective. Of note, no evidence identified in this review compared mammalian cell culture-based inactivated influenza vaccine (IIV-cc) to other influenza vaccines. Following its thorough review, NACI issued a new recommendation on influenza vaccination in adults 65 years of age and older.

**Table 2:** Comparison of the characteristics of influenza vaccine types available for use in adults 65 years of age and older^a^

Characteristics	IIV-HD, IIV-Adj and RIV compared to IIV-SD
Efficacy and effectiveness	IIV-HD, IIV-Adj and RIV appear to have increased vaccine efficacy and effectiveness as compared to IIV-SD. Notably, IIV-HD has the most substantial body of supporting evidence, followed by IIV-Adj and then RIV. The magnitude of relative benefit varied and was not seen in all studies and all seasons. There are few RCTs comparing IIV-HD, IIV-Adj and RIV to IIV-SD and to one another. No RCT compared IIV-Adj with IIV-SD for the outcome of LCI. No definitive conclusion can be reached regarding the superiority of any of these vaccines over one another as there is limited evidence directly comparing IIV-HD, IIV-Adj and RIV against each other. There is limited evidence on newer vaccine technologies (e.g., IIV-cc and RIV). Further evidence is needed on the efficacy and effectiveness of influenza vaccines in subpopulations of adults 65 years of age and older at higher risk of severe influenza-related outcomes and complications, such as advanced-age older adults, individuals living with one or more chronic medical conditions and frail individuals.
Safety	IIV-HD, IIV-Adj and RIV appear to be well-tolerated and safe alternatives to IIV-SD in adults 65 years of age and older. Evidence suggests that there is no difference in safety between IIV-HD, IIV-Adj and RIV based on direct evidence among adults 65 years of age and older. Only a few studies reported data for certain vaccine comparisons (e.g., IIV-Adj vs RIV4). Limited data were available for Guillain-Barré Syndrome.
Economics	IIV-HD and IIV-Adj may be considered cost-effective when compared to IIV-SD under commonly used cost-effectiveness thresholds ( ((17))). There is no economic evidence directly comparing IIV-HD, IIV-Adj, and RIV against each other ( ((16))).
Ethics, equity, feasibility and acceptability	Equity could potentially be increased for older adults at greater risk of severe illness and influenza-related complications if they are given vaccines with higher efficacy. Feasibility from a provider and policymaker perspective may be decreased as enhanced vaccines have higher costs and the level of increased efficacy is uncertain. Acceptability may be increased for high-risk groups due to increased perceived benefits of preferred vaccines in adults 65 years of age and older. Reducing the burden of disease may increase acceptability from the providers’ and policymakers’ perspectives; however, due to a lack of data supporting higher efficacy and potential increased costs, the use of a preferred vaccine may not be as acceptable.

Abbreviations: IIV-Adj, adjuvanted inactivates influenza vaccines; IIV-HD, high-dose inactivated influenza vaccines; IIV-SD, standard-dose inactivated influenza vaccines; LCI, laboratory-confirmed influenza; RCT, randomized controlled trial; RIV, recombinant influenza vaccine; RIV4, recombinant quadrivalent influenza vaccine

^a^ Table taken from *NACI Statement on Seasonal Influenza Vaccine for 2024–2025* ( ((5)))

## Recommendation

NACI recommends that IIV-HD, IIV-Adj or RIV should be offered over other influenza vaccines for adults 65 years of age and older. If a preferred product is not available, any of the available age-appropriate influenza vaccine should be used. (*Strong NACI Recommendation*)

· Where supply of IIV-HD, IIV-Adj or RIV is limited, consideration can be given to prioritizing groups at highest risk of severe outcomes from influenza among adults 65 years of age and older, such as advanced-age older adults (e.g., 75 years of age and older), those with one or more comorbidities, older frail adults and residents of nursing homes and other chronic care facilities.

Summary of evidence

· IIV-HD, IIV-Adj and RIV appear to have increased vaccine efficacy/effectiveness as compared to IIV-SD.

· No definitive conclusion can be reached regarding the superiority of any of these vaccines over one another as there is a limited number of studies directly comparing IIV-HD, IIV-Adj and RIV against each other. Notably, IIV-HD has the most substantial body of supporting evidence, followed by IIV-Adj and then RIV.

· IIV-HD, IIV-Adj and RIV are effective alternatives to IIV-SD, with no identified difference in safety, based on direct evidence among adults 65 years of age and older.

· IIV-HD and IIV-Adj are cost-effective when compared to IIV-SD.

A complete review of evidence and full NACI recommendations are published in the new *NACI Supplemental guidance on influenza vaccination in adults 65 years of age and older* ( ((8))). This supplemental guidance aligns with NACI’s overarching recommendation for influenza vaccination, available in the *NACI Seasonal Influenza Vaccine Statement*, which is that an age-appropriate influenza vaccine should be offered annually to anyone six months of age and older, noting product-specific contraindications **(*Strong NACI Recommendation*)** ( ((5))).

## Conclusion

The available evidence suggests potential advantages associated with IIV-HD, IIV-Adj and RIV compared to IIV-SD; however, the available evidence directly comparing these vaccines to one another is insufficient to establish with certainty that one vaccine consistently outperforms the others. Moreover, data for IIV-HD, IIV-Adj and RIV against IIV-SD demonstrated a comparable safety profile. As the body of evidence exploring whether certain authorized age-appropriate influenza vaccines are better suited for adults 65 years of age and older compared to others continues to grow, NACI will continue to monitor the evolving evidence and will update this guidance as needed. Further evaluation of safety, efficacy and effectiveness data for newer vaccine technologies (e.g., IIV-cc and RIV) as well as vaccine comparisons (pairwise or comparisons between multiple vaccines) between newer influenza vaccines among adults 65 years of age and older are encouraged. Other new and emerging research priorities identified include further evaluation of vaccine efficacy and effectiveness of influenza vaccines stratified by subpopulations of adults 65 years of age and older (e.g., health and frailty status); national-level influenza surveillance data among older adults in Canada; timing of influenza vaccination with respect to duration or waning of protection in adults 65 years of age and older; incorporation and investigation of the impact of community immunity, frailty and longer-term functional outcomes on cost effectiveness; and factors that influence vaccine confidence and acceptability among adults 65 years of age and older in Canada.
